# Effectiveness of national cervical cancer screening programme in Taiwan: 12-year experiences

**DOI:** 10.1038/sj.bjc.6605139

**Published:** 2009-06-16

**Authors:** Y-Y Chen, S-L You, C-A Chen, L-Y Shih, S-L Koong, K-Y Chao, M-L Hsiao, C-Y Hsieh, C-J Chen, and Taiwan Cervical Cancer Screening Task Force

**Affiliations:** 1 Graduate Institute of Epidemiology, College of Public Health, National Taiwan University, Taipei, Taiwan; 2 Genomics Research Center, Academia Sinica, Taipei, Taiwan; 3 Department of Obstetrics and Gynecology, National Taiwan University Hospital, National Taiwan University, Taipei, Taiwan; 4 Bureau of Health Promotion, Department of Health, Executive Yuan, Taipei, Taiwan

**Keywords:** cervical cancer, incidence, mortality, national screening programme, secular trend

## Abstract

**Background:**

We examined cervical cancer incidence before and after nationwide cervical cancer screening was initiated in Taiwan in mid-1995.

**Results:**

The invasive cancer incidence decreased by 47.8% during 1995–2006. The carcinoma *in situ* incidence increased 1.7-fold during 1995–2000, and decreased by 19.6% during 2000–2006.

**Conclusion:**

The Taiwan national programme has significantly decreased invasive cervical cancer.

Cervical cancer has been one of the most serious cancers in Taiwan over the last three decades, accounting for more than 20% of all female cancers before 2001, and for over 10% of female cancer deaths before 1995 (Taiwan Cancer Registry Task Force, 1991–2006). Such reductions following implementation of screening have been well documented (Peto *et al*, 2004; Bray *et al*, 2005). In Taiwan, Pap smear screening was initiated in December 1974 when the Cancer Society of the Republic of China together with gynaecological clinics offered free Pap tests in 1974–1984. Although the programme was advertised through the mass media, only 2.4% of women in the first and 4.5% of women in the second 5 years were screened (Chou and Chen, 1989). The National Labor Insurance began to reimburse the Pap test for all women working in the private and public sectors in 1991, and the national screening programme reimbursed by the National Department of Health was implemented in 1992 with an aim to reimburse Pap tests for 2% of the women population. The National Health Insurance was launched in 1995 (Wen *et al*, 2008), when it started to reimburse annual Pap tests for all women aged 30 years or more (Koong *et al*, 2006). Annual participation gradually increased from 9.4% in 1995 to 27.5% in 2007. But the triennial participation was only 51.0% during 2005–2007, which was lower than those in most regions with organised screening programme. All Pap tests are mandatory to register in the National Cervical Cancer Screening Registry in Taiwan. Any eligible woman who has not attended for screening for more than 3 years is urged to attend the screening by public health nurses of local health centres. Any screened woman affected with a high-grade squamous intraepithelial or worse lesion is personally interviewed by public health nurses to check whether she has received any biopsy, follow-up Pap test, and treatment.

The specific aim of this study is to evaluate whether the disease burden of cervical cancer changed with the implementation of national screening programme in Taiwan, where the screening participation was lower than those in other countries with organised screening programme.

## Materials and methods

In this study, cervical cancer incidence and mortality data were derived from several national registries. Population numbers during 1991–2007 were obtained from National Household Registry profiles (Department of Household Registration, 1991–2007); this was found to be complete (99.3%) according to the 2000 census in Taiwan (Chen and Liu, 2002).

National Cancer Registry in Taiwan was launched in 1979 to collect information of all incident cancer cases from hospitals with 50 or more beds. It was considered a complete and accurate registry with a percentage of cases based on death certificate only (DCO) as low as 3.9% in 2000 and 1.8% in 2005. The DCO percentage for cervical cancer was <1% after 2000, and the percentage of morphological verification of cervical cancer was more than 90% after 2002 (Taiwan Cancer Registry Task Force, 1991–2006). The numbers of incident cases of cervical cancer (International Classification of Diseases for Oncology Field Trial Edition (ICD-O-FT) coded 180 during 1991–2001, and ICD-O Third Edition (ICD-O-3) (Fritz *et al*, 2000) coded C53 after 2002) were obtained from National Cancer Registry profile during 1991–2006. The invasive cancer and carcinoma *in situ* were differentiated according to the ICD-O-FT or ICD-O-3 morphology fifth digit behaviour code for neoplasms. The identification of cancer cell type was according to ICD-O-FT or ICD-O-3 morphology code.

The National Health Insurance was implemented in Taiwan in 1995, covering 90–98% of the general population; it started to reimburse annual conventional Pap smear for women over 30 years of age in 1995. The National Cervical Cancer Screening Registry was also launched in 1995 to evaluate the efficacy and effectiveness of the programme. All Pap smears were reviewed according to 2001 Bethesda system (Solomon *et al*, 2002), and any high-grade lesion or worse detected was automatically referred to the follow-up system. Registration of the Pap test records was mandatory, and 24 million screening records were reported from 100 laboratories from 1995 to 2007. There were 5.6 million eligible women who participated in the screening programme at least once. Annual, triennial, and 5-year numbers of screening participants were derived from the NCCSR profiles during 1995–2007.

## Results

**Figure 1:**
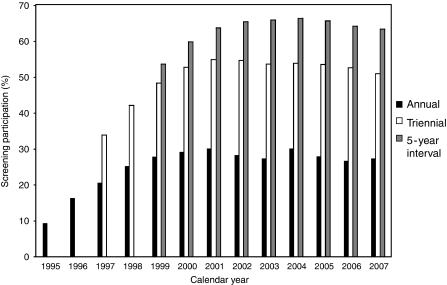
Annual, triennial, 5-year interval, and cumulative cervical cancer screening participation rate for women aged 30 years or more from 1995 to 2007 in Taiwan.

In Taiwan, there were 1800–3000 newly diagnosed cases of invasive cervical cancer cases, and 700–4000 newly diagnosed cases of carcinoma *in situ* in each year during the period from 1991 to 2007. The annual, triennial, and 5-year cervical cancer screening participation in Taiwan from 1995 to 2007 are shown in Figure 1. The annual participation gradually increased from 9.4% in 1995 to 27.5% in 2007, triennial participation increased from 33.9% in 1997 to 51.0% in 2007, and 5-year participation increased from 53.6% in 1999 to 63.5% in 2007. After the implementation of the screening programme in 1995, around 75% eligible women in Taiwan had participated in the programme for at least once by 2007.

**Figure 2:**
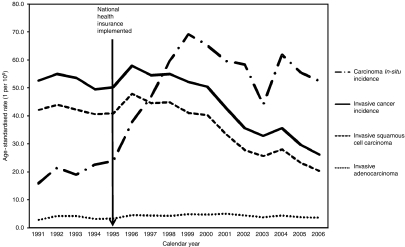
Secular trends of age-standardised rate for cervical cancer mortality, invasive cervical cancer, and cervical carcinoma *in situ* for women aged 30 years or more from 1991 to 2007 in Taiwan.

The secular trends of age-standardised cervical cancer incidence for women aged 30 years or more in Taiwan are shown in Figure 2. Age-adjusted incidence of carcinoma *in situ* per 100 000 women increased from 15.8 in 1991 to 23.9 in 1995. Age-adjusted incidence of invasive cancer increased in the first year after the implementation of screening programme, and then decreased from 57.8 in 1996 to 26.2 in 2006 (54.7% reduction). Age-adjusted incidence of carcinoma *in situ* per 100 000 women increased from 23.9 in 1995 to 69.2 in 1999, and then decreased from 69.2 to 52.4 (24.3% reduction). The age-adjusted incidence of invasive cancer and carcinoma *in situ* rapidly reduced in 2003 and rebounded in 2004 might be due to reduction in screening and confirmatory diagnosis of cervical cancers because of the outbreak of severe acute respiratory syndrome in 2003 in Taiwan.

The incidence of invasive cervical cancer did not decrease significantly in age groups of 30–69 years until 1997 and in age group of 70+ years until 1999. The reduction percentage of incidence of invasive cervical cancer from 1995 to 2006 was 47.5% (from 20.0 to 10.5), 41.9% (from 43.2 to 25.1), 54.8% (from 67.7 to 30.6), 55.5% (from 83.1 to 37.0), and 32.3% (from 77.1 to 52.2), respectively, for age group of 30–39, 40–49, 50–59, 60–69, and 70+ years. The incidence of carcinoma *in situ* increased rapidly after 1995, picked at 1999, and then reduced in most age groups except the age group of 30–39 years.

The main morphological type of invasive cervical cancer in Taiwan was squamous cell carcinoma, but the proportion of adenocarcinoma increased from 5.5% in 1991 to 13.7% in 2006. The incidence of invasive cervical squamous cell carcinoma decreased from 1995 to 2006 showing a reduction percentage of 55.6% (from 16.0 to 7.1), 48.1% (from 34.1 to 17.7), 58.5% (from 56.2 to 23.3), 53.7% (from 67.2 to 31.1), and 31.0% (from 64.2 to 44.3 per 100 000), respectively, for age groups of 30–39, 40–49, 50–59, 60–69, and 70+ years. The incidence of invasive cervical adenocarcinoma decreased from 2000 to 2006 showing a reduction percentage of 15.4% (from 2.6 to 2.2), 31.3% (from 6.4 to 4.4), 21.0% (from 6.2 to 4.9), 31.9% (from 4.7 to 3.2), and 7.9% (from 3.8 to 3.5 per 100 000), respectively, for age groups of 30–39, 40–49, 50–59, 60–69, and 70+ years.

## Discussion

The study provided evidence that national organised screening programme significantly reduced cervical cancer risk in Taiwan. We used several complete and accurate national databases to assess efficacy. The significant rapid and gradual reduction observed in this study was obviously attributable to the programme. Organised cervical cancer screening is well-known to effectively reduce invasive cancer incidence in developed countries, and consistent findings were observed in this study. There was 48.0% reduction in invasive cancer incidence from 1995 to 2006 in women with an average triennial screening participation of 53.4% after 2000. Compared with the slow reduction in incidence before the national screening programme, the rapid reduction after the screening programme provides evidence for its efficacy.

The screening may decrease the incidence of invasive cancer by detecting precancerous lesions. The incidence of invasive cancer in regions with more than 70% triennial screening participation reduced at least 35% after 10 years of organised screening, but 52% triennial participation in Taiwan only reduced 28.9% (Anttila *et al*, 2004). It is also worth to note that under the low participation, the incidence of cervical carcinoma *in situ* also significantly reduced in all age groups older than 35 years.

In this study, the screening programme significantly reduced the incidence of squamous cell carcinoma and adenocarcinoma in all age groups. A recent study of cervical adenocarcinoma incidence in 13 European countries (Bray *et al*, 2005) found a decline in the United Kingdom, Denmark, and Sweden in the 1990s, primarily among women aged 30 years or more, and an increase in other countries. Improvements of Pap smear technique, such as the extended tip spatula or the endocervical brush, have aided general practitioners to collect inner cervix cells (Buntinx and Brouwers, 1996). However, the reduction in cervical adenocarcinoma incidence was not observed in all regions where extended tip spatula was generally used (Visioli *et al*, 2004). The awareness of adenocarcinoma *in situ* as precancerous lesions facilitated early detection of malignant lesions (Mitchell *et al*, 2004) with consequently increased incidence of adenocarcinoma. In Taiwan, the incidence of cervical adenocarcinoma increased for several years after the screening programme was implemented, and then reduced slightly. Whether this significantly reduces cervical adenocarcinoma incidence needs a longer time to determine.
